# Characterization of the model for experimental testicular teratoma in 129/SvJ-mice

**DOI:** 10.1038/sj.bjc.6690334

**Published:** 1999-04

**Authors:** J Sundström, L J Pelliniemi, T Kuopio, E Veräjänkorva, K Fröjdman, V Harley, E Salminen, P Pöllänen

**Affiliations:** Departments of Anatomy, Oncology, Pathology, Obstetrics and Gynaecology and Laboratory of Electron Microscopy, University of Turku, FIN-20520 Turku, Finland; Department of Anatomy, University of Helsinki, PO Box 9, 00014 Helsinki, Finland; Howard Florey Institute of Experimental Physiology and Medicine, University of Melbourne, Parkville, Vic, 3052, Australia

**Keywords:** animal model, mouse, testicular teratoma, characterization

## Abstract

An animal model of experimental testicular teratoma has been established to study how a teratoma affects the host testis and how the host testis reacts against the teratoma. 129/SvJ-mice were used as experimental animals. To induce the experimental testicular teratoma, male gonadal ridges from 12-day-old 129/SvJ-mouse fetuses were grafted into the testes of adult mice for 1–12 weeks. The developing tumour was analysed by light and electron microscopy and by immunocytochemical localization of transcription factors SOX9 and c-kit, glial fibrillary acidic protein (GFAP) and type IV collagen. Testicular teratoma was observed in 36 out of 124 testes with implanted fetal gonadal ridges (frequency 29%). One spontaneous testicular teratoma was observed in this material from 70 male mice (1.5%). One week after implantation intracordal clusters of cells were seen in embryonic testicular cords of the graft as the first sign of testicular teratomas. Four weeks after implantation the embryonic testicular cords had totally disappeared from grafts with teratomas, and the tumour tissue had enlarged the testis and invaded the interstitium of the host testis. It consisted of solitary pieces of immature cartilage as well as of glial cells and of primitive neuroepithelium. Six to eight weeks after implantation the tumour tissue had expanded so that the enlarged testis could be detected by macroscopic enlargement of the scrotum. The testicular tissue of the host had practically disappeared, and only solitary disrupted seminiferous tubules of the host were seen surrounding the teratoma. Neuroepithelial structures of some teratomas cultured for 8 weeks had cells with a granular nucleus as a sign of obvious apoptosis. Eleven to 12 weeks after implantation the growth of the teratoma had stopped, and the histology corresponded to that of a mature cystic teratoma. GFAP, SOX9 and type IV collagen were strongly positive in some parts of the tumours cultured for 4 and 8 weeks, while only occasional c-kit-positive areas were observed in tumours cultured for 8 weeks. As conclusions: (1) the metastasizing capacity of the experimental testicular teratoma is very low during 12 weeks, but the behaviour of the tumour in the testicular tissue of the graft is invasive; (2) the growth of experimental testicular teratomas cease 6–8 weeks after implantation of the fetal gonadal ridges with the obvious apoptosis of the immature tissue components; (3) the model of experimental testicular teratoma in the mouse is suitable for studying how the teratoma affects the host testis and how the host testis reacts to teratoma. © 1999 Cancer Research Campaign


*British Journal of Cancer* (1999) **80**(1/2), 149–160
© 1999 Cancer Research Campaign
Article no. bjoc.1998.0334

### Characterization of the model for experimental testicular teratoma in 129/SvJ-mice

**J Sundström[1–3], LJ Pelliniemi[3], T Kuopio[4], E Veräjänkorva[1], K Fröjdman[6], V Harley[7], E Salminen[2] and P Pöllänen[1,5]**

Departments of [1]Anatomy, [2]Oncology, [4]Pathology, [5]Obstetrics and Gynaecology and [3]Laboratory of Electron Microscopy, University of Turku, FIN-20520 Turku, Finland; [6]Department of Anatomy, University of Helsinki, PO Box 9, 00014 Helsinki, Finland; [7]Howard Florey Institute of Experimental Physiology and Medicine, University of Melbourne, Parkville, Vic., 3052, Australia

**Summary** An animal model of experimental testicular teratoma has been established to study how a teratoma affects the host testis and how the host testis reacts against the teratoma. 129/SvJ-mice were used as experimental animals. To induce the experimental testicular teratoma, male gonadal ridges from 12-day-old 129/SvJ-mouse fetuses were grafted into the testes of adult mice for 1–12 weeks. The developing tumour was analysed by light and electron microscopy and by immunocytochemical localization of transcription factors SOX9 and c-kit, glial fibrillary acidic protein (GFAP) and type IV collagen. Testicular teratoma was observed in 36 out of 124 testes with implanted fetal gonadal ridges (frequency 29%). One spontaneous testicular teratoma was observed in this material from 70 male mice (1.5%). One week after implantation intracordal clusters of cells were seen in embryonic testicular cords of the graft as the first sign of testicular teratomas. Four weeks after implantation the embryonic testicular cords had totally disappeared from grafts with teratomas, and the tumour tissue had enlarged the testis and invaded the interstitium of the host testis. It consisted of solitary pieces of immature cartilage as well as of glial cells and of primitive neuroepithelium. Six to eight weeks after implantation the tumour tissue had expanded so that the enlarged testis could be detected by macroscopic enlargement of the scrotum. The testicular tissue of the host had practically disappeared, and only solitary disrupted seminiferous tubules of the host were seen surrounding the teratoma. Neuroepithelial structures of some teratomas cultured for 8 weeks had cells with a granular nucleus as a sign of obvious apoptosis. Eleven to 12 weeks after implantation the growth of the teratoma had stopped, and the histology corresponded to that of a mature cystic teratoma. GFAP, SOX9 and type IV collagen were strongly positive in some parts of the tumours cultured for 4 and 8 weeks, while only occasional c-kit-positive areas were observed in tumours cultured for 8 weeks. As conclusions: (1) the metastasizing capacity of the experimental testicular teratoma is very low during 12 weeks, but the behaviour of the tumour in the testicular tissue of the graft is invasive; (2) the growth of experimental testicular teratomas cease 6–8 weeks after implantation of the fetal gonadal ridges with the obvious apoptosis of the immature tissue components; (3) the model of experimental testicular teratoma in the mouse is suitable for studying how the teratoma affects the host testis and how the host testis reacts to teratoma.

**Keywords:** animal model; mouse; testicular teratoma; characterization

In the human testis, germ cell tumours form approximately 90% of all testicular neoplasms (Mostofi and Price, 1973). They arise from the germinal epithelium of seminiferous tubules. Testicular germ cell tumours are the most common malignant tumours in young men in the Western world and the incidence is increasing (Adami et al, 1994; Gilliland and Key, 1995; Zheng et al, 1996) simultaneously with the decreasing sperm quality (Carlsen et al, 1992). The prognosis of testicular tumours varies widely according to the clinical stage and tumour type (Klepp et al, 1990; Ro et al, 1991).

In human it is difficult to follow the initial steps of testicular tumour development. Therefore it is useful to have an animal model for this purpose. This kind of a model can also be used for studying the effects of the testicular tumour on the surrounding host testis. Some sub-lines of inbred strain 129-mice have spontaneous testicular teratomas (Stevens, 1967*a*, 1973), and the incidence of testicular teratomas in these mice increases considerably by implanting male fetal gonadal ridges of 12-day-old 129/Sv-mice to the testicular interstitial tissue of adult mice from the same strain (Stevens, 1964). The mouse strain used in our studies is the 129/SvJ from the Jackson Laboratory (Bar Harbor, ME, USA). The method of gonadal ridge implantation has been used in this study to induce testicular teratomas in 129/SvJ-mice. Several mutations affect susceptibility to teratomas in 129/Sv-mice (Stevens and Mackensen, 1961; Stevens 1973, Matin et al, 1998).

The aim of this study is to characterize with modern cell biological methods the development of the testicular teratoma and the morphology of the surrounding host testis 1–12 weeks after the implantation of fetal gonadal ridge. The markers for immunocytochemistry have been chosen to observe the changes in main cell populations of embryonic testicular cords and the seminiferous tubules of the host testis, that is SOX9 for Sertoli cells and c-kit for germ cells. Glial fibrillary acidic protein (GFAP) localizes the neural tissue components of the testicular teratoma and type IV collagen demonstrates the border between the host testis and the growing teratoma.

*Received 27 July 1998*
*Revised 16 October 1998*
*Accepted 4 November 1998*

*Correspondence to*: J Sundström, Department of Anatomy, University of Turku, Kiinamyllynkatu 10, FIN-20520 Turku, Finland

### MATERIALS AND METHODS

### Antibodies

The purified polyclonal rabbit anti-cow GFAP (isolated from cow spinal cord) immunoglobulin fraction of rabbit antiserum (Dako, Glostrup, Denmark) and the purified polyclonal rabbit anti-human c-kit (corresponding to residues 958–976 within C-terminal domain of human c-kit) immunoglobulin fraction of rabbit antiserum (Santa Cruz Biotechnology, Santa Cruz, CA, USA) were used. In addition antibodies to type IV collagen (Foidart et al, 1980) recognizing at least the α1/α2(IV) chains (Fröjdman et al, 1998) and antibodies for the last 24 amino acids of full length human SOX9 (VR Harley, University of Melbourne, Parkville, Australia) raised in rabbits were used.

### Immunocytochemistry

The tumour-containing tissues were removed and frozen immediately in liquid nitrogen. Sections of 6–8 μm in thickness were cut in a cryostat and dried on slides in air. To localize GFAP, c-kit and type IV collagen from tumours the sections were fixed in cold (–20°C) acetone for 7 min. The acetone was allowed to evaporate before storing the sections at –20°C. To localize SOX9 in tumours the sections were fixed in periodate–lysine–paraphormaldehyde (PLP) (McLean and Nakane, 1974) in room temperature (RT) for 10 min. The sections were soaked in 0.05 mol $l^{-1}$ Tris–hydroxymethyl aminomethane–HCl buffer, pH 7.6 (Tham-HCl) containing 0.9% sodium chloride (TBS) at RT for 10 min to allow stabilization of the temperature before incubations. Non-specific binding sites were blocked by incubating the tissues in 1.6% goat serum (Vector, Burlingame, CA, USA) in TBS for 30 min. The excess of goat serum in TBS was removed from the slides and the sections were incubated with polyclonal rabbit anti-cow GFAP, polyclonal rabbit anti-human c-kit, polyclonal rabbit anti-rat type IV collagen and polyclonal rabbit anti-human SOX9 antibodies at +4°C overnight [diluted 1:8000, 1:100, 1:2000 and 1:2000 in 0.1% bovine serum albumin (BSA, Sigma, Steinheim, Germany) in TBS respectively]. After washing in TBS, the avidin–biotin complex method using biotinylated goat anti-rabbit secondary antibody was employed using the ABC Elite kit (Vector, Burlingame, CA, USA) as previously described (Hsu et al, 1981). Diaminobenzidine tetrahydrochloride (Sigma, Steinheim, Germany) was used as a chromogen. Negative control slides repeated all steps, replacing the primary antibody by rabbit immunoglobulin fraction. The slides were then dehydrated and embedded (Depex®, Gurr). The sections were examined and photographed in bright field using a Leitz Diaplan microscope (Ernst Leitz Wetzlar, Germany).

### Animals and induction of tumours

129/SvJ-mice (Stock # 000691, inbred) from the Jackson Laboratory (Bar Harbor, ME, USA) were used as experimental animals. The number of mice with intratesticularly implanted fetal gonadal ridges cultured for various times were as follows: for 1 week ($n$ = 19), for 2 weeks ($n$ = 7), for 3 weeks ($n$ = 7), for 4 weeks ($n$ = 8), for 6–8 weeks ($n$ = 19) and for 9–12 weeks ($n$ = 10). One male was housed with one female per cage for breeding for 24 h, after which the females were examined for a vaginal plug. On the day on which the plug was found, the age of the embryos was considered to be 0 days. Embryos were obtained at the age of 12 days. The females were killed with carbon dioxide ($CO_2$). A medial ventral incision was made and the uterus was opened. The embryos were dissected out from the uterus and fetal membranes under dissecting microscope and killed with immediate decapitation. The abdominal contents of the embryos were removed under dissecting microscope and gonadal ridges were dissected out from the posterior abdominal wall, separated from mesonephros and placed in fresh Hank's medium (+ 4°C), for 3–4 h. The sex of the indifferent gonads was identified by X chromatin staining in amniotic membrane (Orcein®, Fluka). The testes of anaesthetized (Hypnorm®: fentanyl citrate 0.315 mg $ml^{-1}$ and fluanisone 10 mg $ml^{-1}$, Janssen-Cilag Ltd, Saunderton, High Wycombe, Buckinghamshire; Dormicum®: midazolam 1 mg $ml^{-1}$, F. Hoffmann-La Roche AG, Basel, Schweiz, Aqua sterilisata; Kabi Pharmacia AB, Sweden; 1:1:2 respectively) adult male mice from the same strain were pushed to the abdomen through the inguinal canal and exposed through medial ventral incision. Gonadal ridges from male embryos were implanted into adult testes with a glass capillary. The animals were killed with $CO_2$ 1–12 weeks after implantation of the gonadal ridges. The testes were removed and frozen immediately in liquid nitrogen. In this strain the frequency of spontaneous testicular teratomas is low, under 2%, and spontaneous testicular teratomas in strain 129 mice can be easily detected by visual examination of exposed testis in animals after 1 week of age (Stevens, 1984). Thus, possible spontaneous testicular teratomas can be excluded in host animals before gonadal ridge implantation.

### Conventional light and electron microscopy

For light microscopy the testes with teratomas were taken from mice freshly sacrificed with $CO_2$, cut into two halves and fixed for 1 day in Bouin's fixative. The tissues were kept in 70% ethanol for another day, embedded in paraffin and cut into 5–10 μm sections. The sections were stained with haematoxylin and eosin.

For electron microscopy the testes with teratomas were immersion-fixed with 5% glutaraldehyde (Merck Darmstadt, Germany) in 0.16 mol $l^{-1}$ s-collidine-HCl buffer (pH 7.4) and post-fixed with potassium ferrocyanide-osmium fixative (Karnovsky, 1971). The tissues were embedded in epoxy resin (Glycidether 100, Merck, Darmstadt, Germany) and sectioned for light and electron microscopy. Ultrathin sections were stained with 12.5% uranyl acetate (Stempak and Ward, 1964) and 0.25% lead citrate (Venable and Coggeshall, 1965) and examined in a Jeol JEM-100C electron microscope. For light microscopy, 1-μm-thick sections were stained with 0.5% toluidine blue.

### Testis extracts and Western blot analysis

Testes from a 129/SvJ-mouse with bilateral testicular teratomas cultured for 7 weeks were freshly removed, pooled, weighed and homogenized in a glass homogenizer (1 g tissue for every 3 ml distilled water) supplemented with 1 μg $ml^{-1}$ aprotinin (Sigma, St Louis, MO, USA) and soybean trypsin inhibitor (Sigma, St Louis, MO, USA) to avoid proteolysis. The homogenates were centrifuged at 250 ***g*** for 15 min. The supernatant was collected and centrifuged at 10 000 ***g*** for 30 min to separate soluble material from membranes and debris. Salts were removed from the second supernatant in a Sephadex G-25 PD-10 column (1.5 × 5 cm, Pharmacia, Uppsala, Sweden) chromatography and freeze-dried. Protein in the samples was measured and diluted to 1 μg $μl^{-1}$ in 2 × Laemmli solution [1% sodium dodecyl sulphate (SDS), 10% glycerol, 0.01% bromophenol blue and 2% β-mercaptoethanol in 50 mM Tris buffer, pH 6.8]. The samples were boiled for 5 min.

 © Cancer Research Campaign 1999

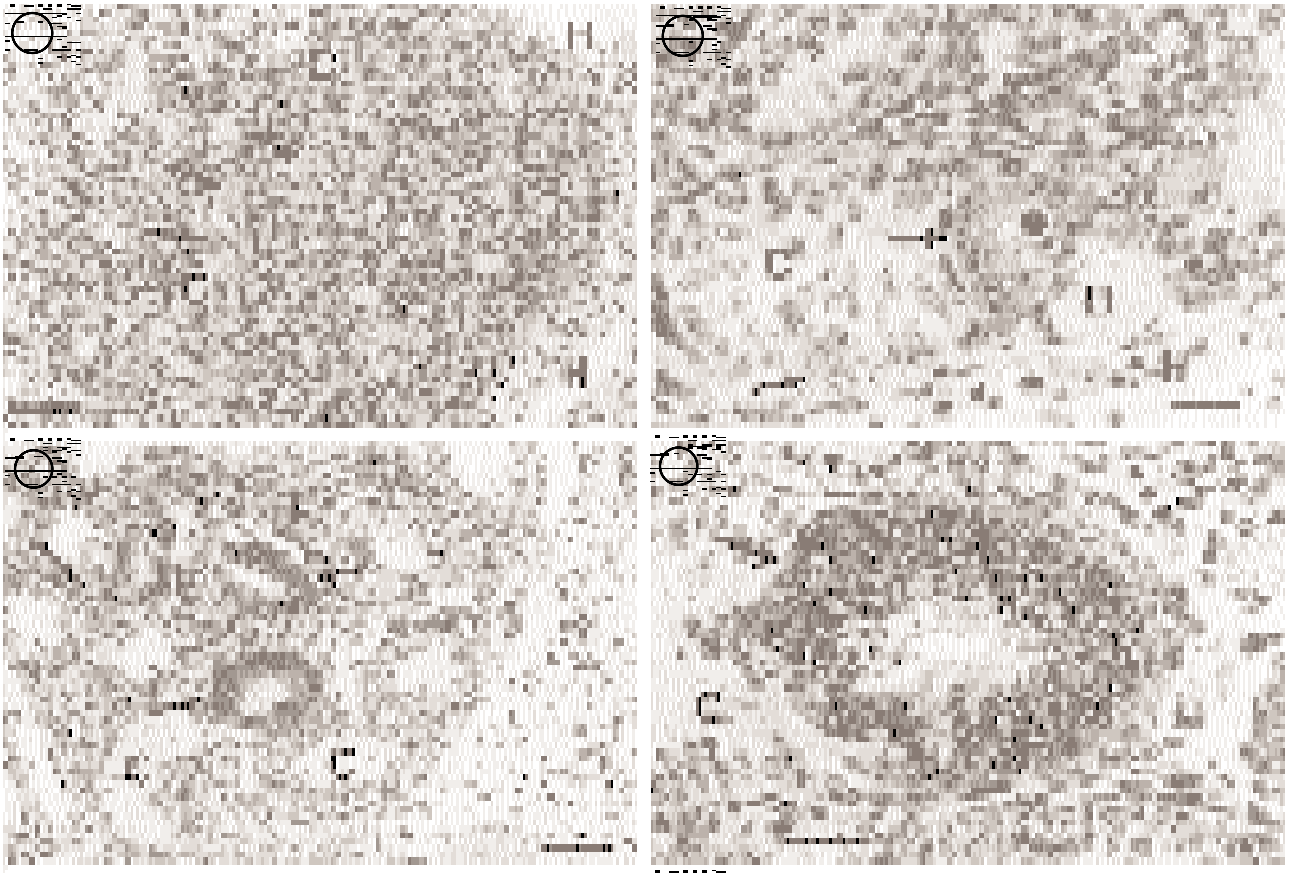

**Figure 1** Light micrograph of the male gonadal ridge of a 12-day-old fetal 129/SvJ-mouse implanted to an adult mouse testis from the same strain for 1 week. Intracordal clusters of atypical cells (arrows) are seen in embryonic testicular cords (C). H = host testis. Bar = 80 μm
**Figure 2** Light micrograph of the male gonadal ridge of a 12-day-old fetal 129/SvJ-mouse implanted to an adult mouse testis from the same strain for 1 week. Intracordal clusters of cells (arrows) with a large nucleus and abundant cytoplasm are seen in higher magnification as in Figure 1. C = embryonic testicular cord, H = host testis, B = blood vessel. Bar = 30 μm
**Figure 3** Light micrograph of the male gonadal ridge of a 12-day-old fetal 129/SvJ-mouse implanted to an adult mouse testis from the same strain for 2 weeks. The originally intracordal clusters of cells have grown (arrows) and ruptured the embryonic testicular cords (C) and spread to the interstitium (I) of the embryonic testicular graft. Bar = 80 μm
**Figure 4** Higher magnification from Figure 3. Note the primitive epithelium (arrow) in the embryonic testicular cord (C). Bar = 30 μm

Denaturating 7.5% SDS-polyacrylamide mini-gels were prepared and 10–20 μl of the extracted sample were loaded to the wells. Low-molecular weight markers (Pharmacia LKB, Uppsala, Sweden) were run parallel to the samples. Gels were run at a 150 mA current and, after electrophoresis, proteins were transferred in to nitrocellulose filter for 60 min as described by Towbin et al (1979). The nitrocellulose filter was stained with Ponceau S and each separate line was cut off. Strips were blocked with 2% BSA in Tris-buffered saline with Tween 20 (TBST) for 30 min and then incubated for 1 h at RT with antibodies against GFAP, SOX9, c-kit and type IV collagen [diluted 1:300, 1:300, 1:100 and 1:300 in 0.1% BSA (Sigma, Steinheim, Germany) respectively in TBST]. After incubation, strips were washed three times with TBST and then incubated for 1 h at RT with biotinylated goat anti-rabbit secondary antibody followed by avidin–biotin complex method of the ABC Elite kit (Vector, Burlingame, CA, USA) as described by Hsu et al (1981). Strips were washed again with TBST and then allowed to react with 0.6 mg $ml^{-1}$ diaminobenzidine tetrahydrochloride (Sigma, St Louis, MO, USA) and 0.003% hydrogen peroxide in 0.005 M Tris (pH 7.6) for 10 min. Reactions were stopped with phosphate-buffered saline (PBS) containing 0.2% sodium azide and strips were blotted dry before photography.

### Preabsorption of SOX9 antibody

The peptide consisting of the last 24 amino acids of full length human SOX9 (VR Harley, University of Melbourne, Parkville, Australia) was used as blocking peptide. The lyophilized peptide was resuspended in distilled water (the concentration: 24 μg $\mu l^{-1}$). For neutralization the SOX9 antibody (VR Harley, University of Melbourne, Parkville, Australia) diluted in 1:10 000 in 0.1% BSA (Sigma, Steinheim, Germany) in TBS was incubated with a 50-fold excess of SOX9-peptide overnight at +4°C. Following neutralization, the avidin–biotin complex method was used as described by Hsu (1981).

### Statistical analysis

The differences in the numbers of samples with embryonic cords present at various times after implantation were analysed using Fisher's exact test.

© Cancer Research Campaign 1999 

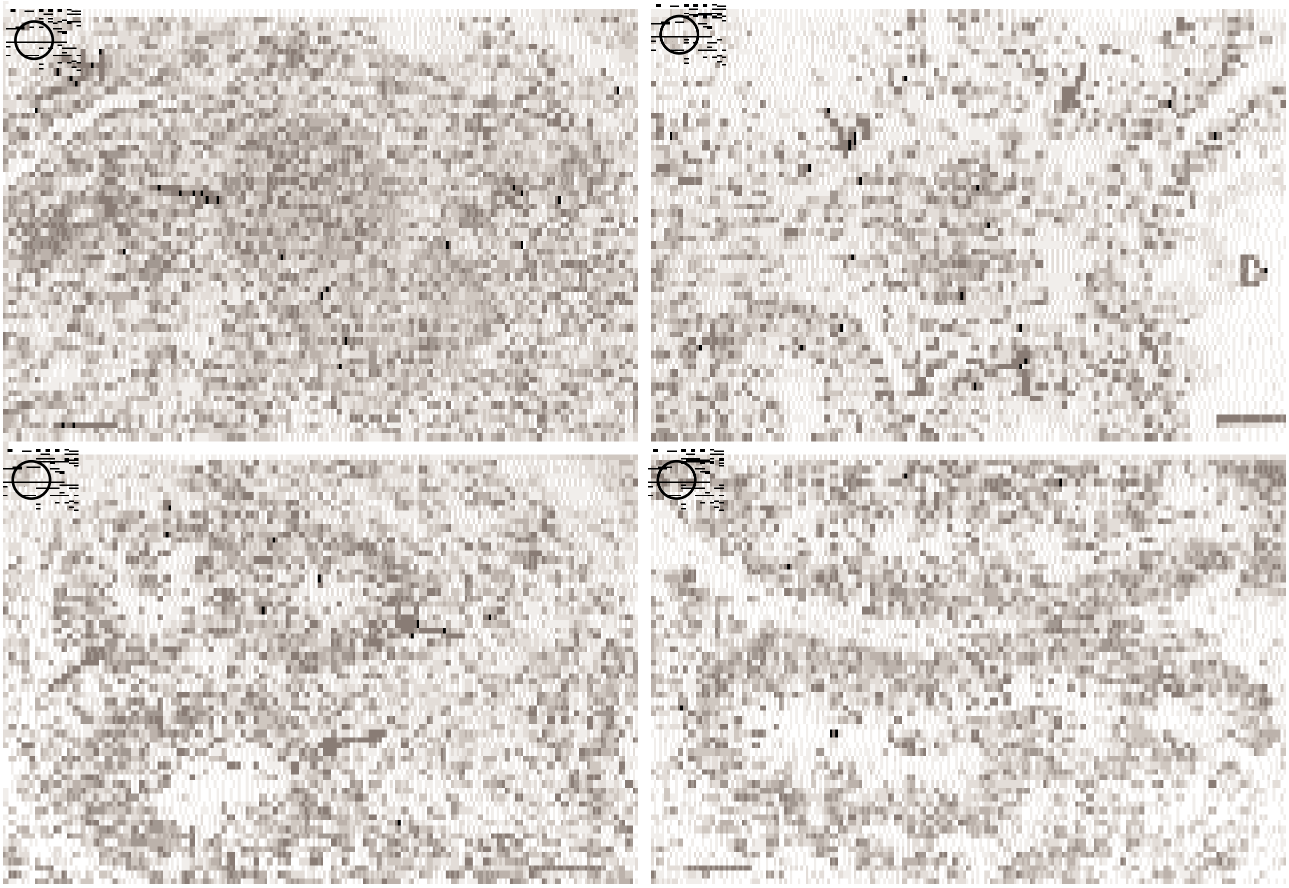

**Figure 5** Light micrograph of the male gonadal ridge of a 12-day-old fetal 129/SvJ-mouse implanted to an adult mouse testis from the same strain for 3 weeks. A piece of cartilage (arrow) is seen in the graft as a sign of teratomatous development. H = host testis. Bar = 80 μm
**Figure 6** Light micrograph of the male gonadal ridge of a 12-day-old fetal 129/SvJ-mouse implanted to an adult mouse testis from the same strain for 4 weeks. The glial component of teratoma has spread to the interstitium of the host testis (arrows) and the degeneration of the host seminiferous tubules (D) has begun. Bar = 50 μm
**Figure 7** Light micrograph of the male gonadal ridge of a 12-day-old fetal 129/SvJ-mouse implanted to an adult mouse testis from the same strain for 4 weeks. Small aggregates of immature neuroepithelium (arrows) can be observed as a sign of primitive neuroectodermal differentiation. Bar = 50 μm
**Figure 8** Light micrograph of the male gonadal ridge of a 12-day-old fetal 129/SvJ-mouse implanted to an adult mouse testis from the same strain for 4 weeks. Frequent mitoses are seen in the primitive neural epithelium (arrows). Bar = 50 μm

### RESULTS

### General observations

The 12-day-old fetal gonadal ridge was implanted to both testes of 54 adult 129/SvJ-mice and to one testis (cultured for 1 week) of 16 129/SvJ-mice. Testicular teratoma was observed in 36 out of 124 testes with implanted fetal gonadal ridges (frequency 29%). Only one unilateral spontaneous testicular teratoma was observed among these 70 mice (frequency 1.5%).

### The grafts and host testis

*The grafts implanted for 1–4 weeks*

The frequency of induced teratomas has been reported by giving the number of teratomas per number of host testes in each group.

One week after implantation intracordal clusters of cells with large nucleus and abundant cytoplasm were seen in embryonic testicular cords (Figures 1 and 2). In the centre of some clusters a small lumen was seen. Variable local damage was observed in the seminiferous tubules surrounding the graft. The frequency of teratomas was 6/22.

Two weeks after implantation the originally intracordal clusters had grown considerably to form areas of columnar epithelium with frequent mitoses. They had ruptured the original embryonic testicular cords (Figures 3 and 4). Unorganized clusters of similar cells were also seen in the interstitium of the graft. The frequency of teratomas was 5/14.

Three weeks after implantation the tumours had grown in size. In addition to epithelial structures, tissue pieces of immature cartilage were seen in some tumours (Figure 5). The frequency of teratomas was 3/14.

Four weeks after implantation the first macroscopic signs of testicular teratomas were observed. When the testes were exposed in laparotomy, a distinct oedema of the testes with teratomas was observed. At this stage, the embryonic testicular cords had totally disappeared from the grafts with teratomas while the embryonic cords were present in grafts cultured for shorter times ($P < 0.05$, Fisher's exact test). At this stage, using light microscopy, the teratoma had invaded over half of the testicular volume, and the teratoma had spread to interstitium of the host testis (Figure 6). As a result, the degeneration of the seminiferous tubules of the host testis was seen. The tissues of neural origin formed the main tissue

© Cancer Research Campaign 1999

**Table 1** The characteristics of the experimental testicular teratomas cultured for 1–4 weeks after implantation of fetal gonadal ridges to adult mouse testes

| Culture time of intratesticular grafts (weeks) | Number of testes with teratomas/ testes per group | Tissue components in teratomas | Size of teratomas |
|---|---|---|---|
| 1 | 6/22 | Undifferentiated clusters of cells | Intracordal clusters of undifferentiated cells in embryonic testicular cords |
| 2 | 5/14 | Rapidly proliferating columnar epithelium and undifferentiated cells | Rupture of embryonic testicular cords, spread of teratoma to interstitium of the embryonic testicular graft |
| 3 | 3/14 | Epithelial structures, pieces of cartilage | Most of the graft consists of teratoma |
| 4 | 7/16 | Neural main component with glial cells, pieces of cartilage, respiratory epithelium, immature teratoma with areas of rapidly proliferating neuroepithelium | The embryonic testicular cords have totally disappeared from the graft, the invasion of the teratoma to interstitium of the host testis, the teratoma has invaded over half of the testicular volume |

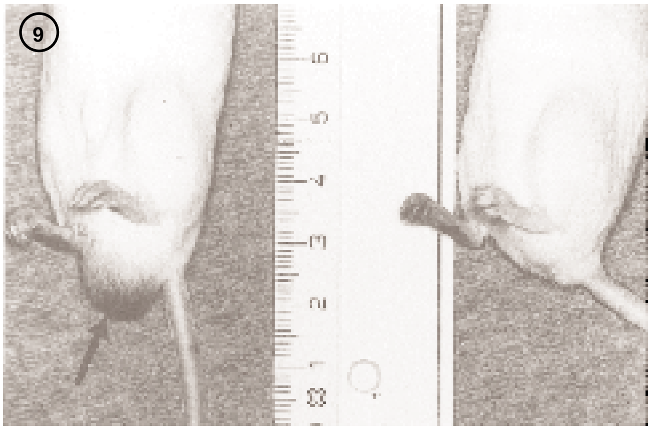

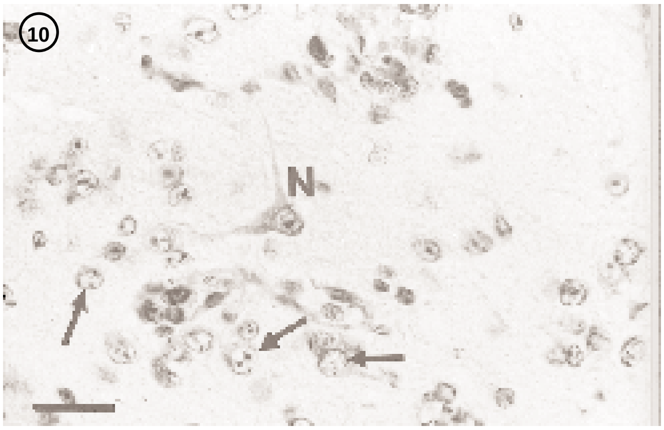

**Figure 9** A photograph of an adult male 129/SvJ-mouse with experimental testicular teratoma. The scrotum has enlarged (arrow) as a sign of testicular teratoma. A control animal of the same age without testicular teratoma to the right. The teratoma has grown from the male gonadal ridge of a 12-day-old fetal 129/SvJ-mouse implanted to an adult mouse testis from the same strain for 8 weeks
**Figure 10** Light micrograph of the male gonadal ridge of a 12-day-old fetal 129/SvJ-mouse implanted to an adult mouse testis from the same strain for 8 weeks. The main component of this teratoma is composed of well differentiated glial cells (arrows). N = neuron. Bar = 30 μm components of teratomas. The histology corresponded to that of an immature teratoma with rapidly proliferating clusters of primitive neuroectodermal tissue (Figures 7 and 8). The characteristics of experimental testicular teratomas cultured for 1–4 weeks have been summarized in Table 1. The frequency of teratomas was 7/16.

### *The grafts implanted for 6–12 weeks*

The frequency of induced teratomas has been reported by giving the number of teratomas per number of implanted host testes in each group.

Six to 8 weeks after implantation the growth of teratoma could be seen macroscopically by enlargement of the scrotum (Figure 9). In addition to tissues of neural origin (Figure 10), the differentiation of the teratoma into a variety of normal embryonic tissue components was observed (Figures 11–14). The vascularization of the tumour was abundant. The histology corresponded to that of a teratoma with local immature areas. The immaturity was related to neuroepithelium. Interestingly, the neuroepithelial structures of some teratomas cultured for 8 weeks had cells with granular nucleus as an obvious sign of undergoing apoptosis (Figure 15).

© Cancer Research Campaign 1999

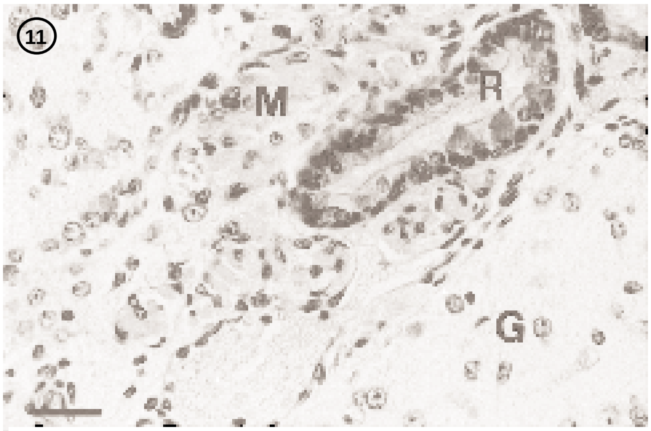

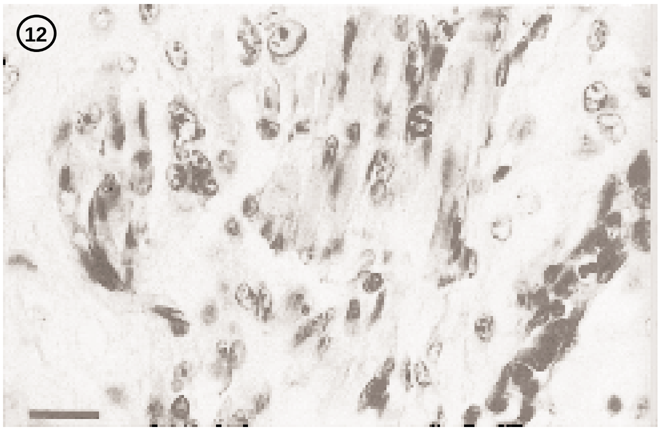

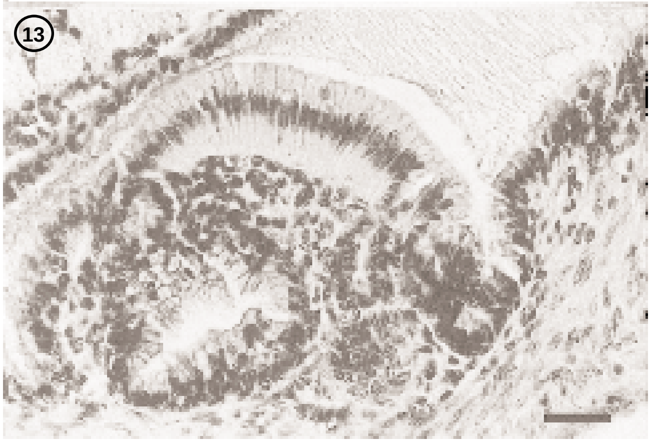

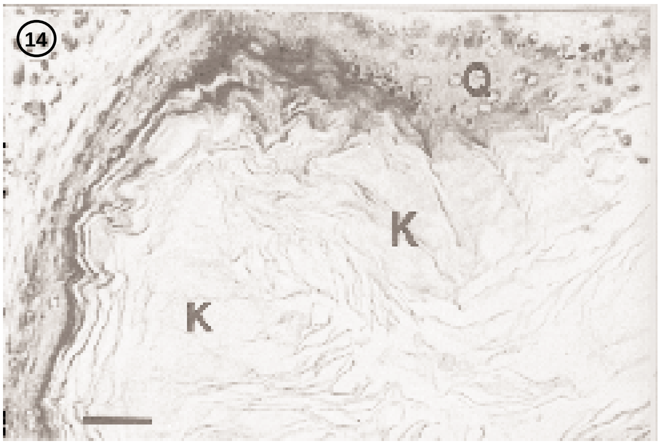

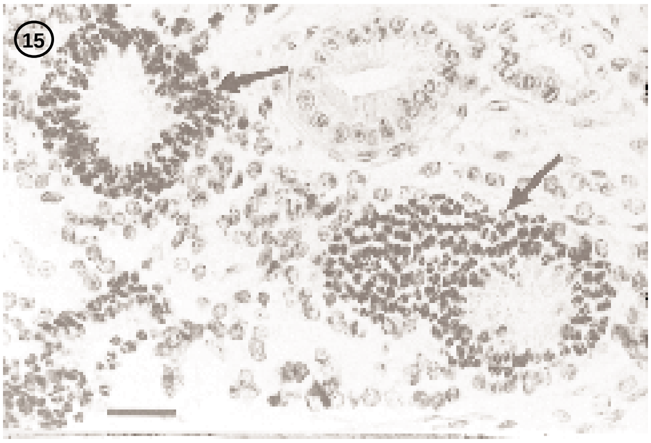

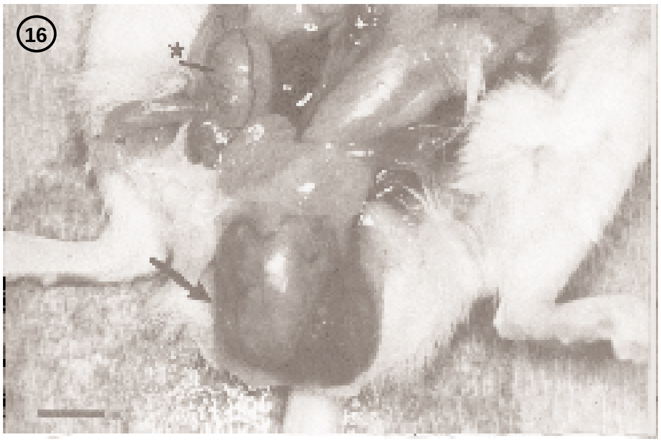

**Figure 11** Light micrograph of the male gonadal ridge of a 12-day-old fetal 129/SvJ-mouse implanted to an adult mouse testis from the same strain for 8 weeks. Glial cells (G), smooth muscle (M) and respiratory-like epithelium (R) are seen in this tumour. Bar = 30 μm
**Figure 12** Light micrograph of the male gonadal ridge of a 12-day-old fetal 129/SvJ-mouse implanted to an adult mouse testis from the same strain for 8 weeks. Pieces of striated muscle (S) are seen in this tumour. Bar = 20 μm
**Figure 13** Light micrograph of the male gonadal ridge of a 12-day-old fetal 129/SvJ-mouse implanted to an adult mouse testis from the same strain for 8 weeks. Polymorphic epithelial structures are seen in this teratoma. Bar = 40 μm
**Figure 14** Light micrograph of the male gonadal ridge of a 12-day-old fetal 129/SvJ-mouse implanted to an adult mouse testis from the same strain for 8 weeks. The cavity is lined with stratified squamous epithelium (Q) and filled with sheets of keratin (K). Bar = 40 μm
**Figure 15** Light micrograph of the male gonadal ridge of a 12-day-old fetal 129/SvJ-mouse implanted to an adult mouse testis from the same strain for 8 weeks. The tubular structures (arrows) with nuclear fragmentation suggest the beginning of spontaneous apoptosis in the immature neuroepithelium. Bar = 30 μm
**Figure 16** A photograph of an adult male 129/SvJ-mouse with experimental testicular teratoma. A large teratoma is seen in the left testis (arrow), while the right testis (asterisk) is of normal size. The teratoma has grown from the male gonadal ridge of a 12-day-old fetal 129/SvJ-mouse implanted to an adult mouse testis from the same strain for 11 weeks. Bar = 5 mm

There were only few remnants of the testicular tubules of the host under the tunica albuginea. The basement membrane of these severely degenerated seminiferous tubules was thickened, and several residual bodies could be seen in these seminiferous tubules by electron microscopy (Figure 17). The frequency of teratomas was 9/38.

 *© Cancer Research Campaign 1999*

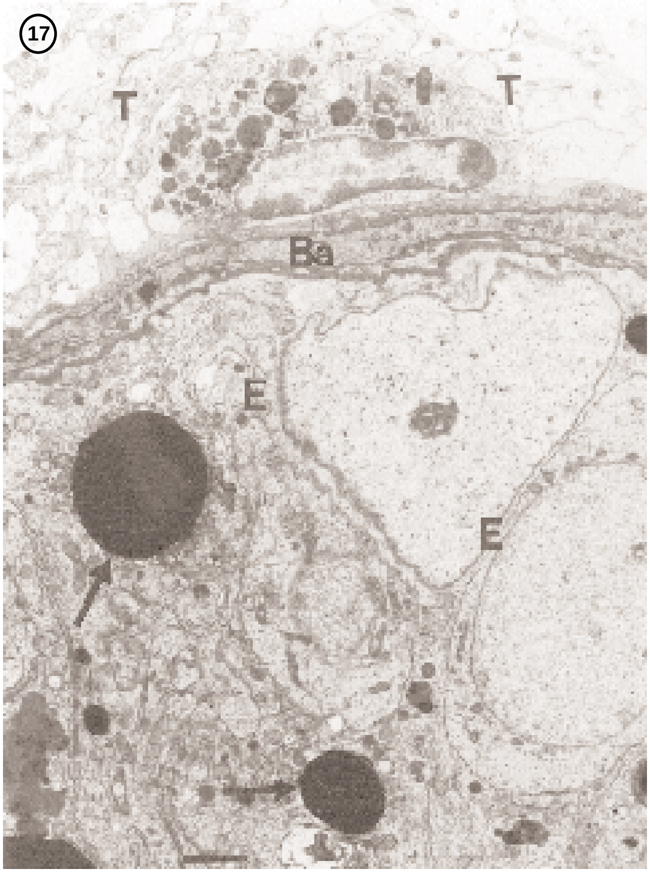

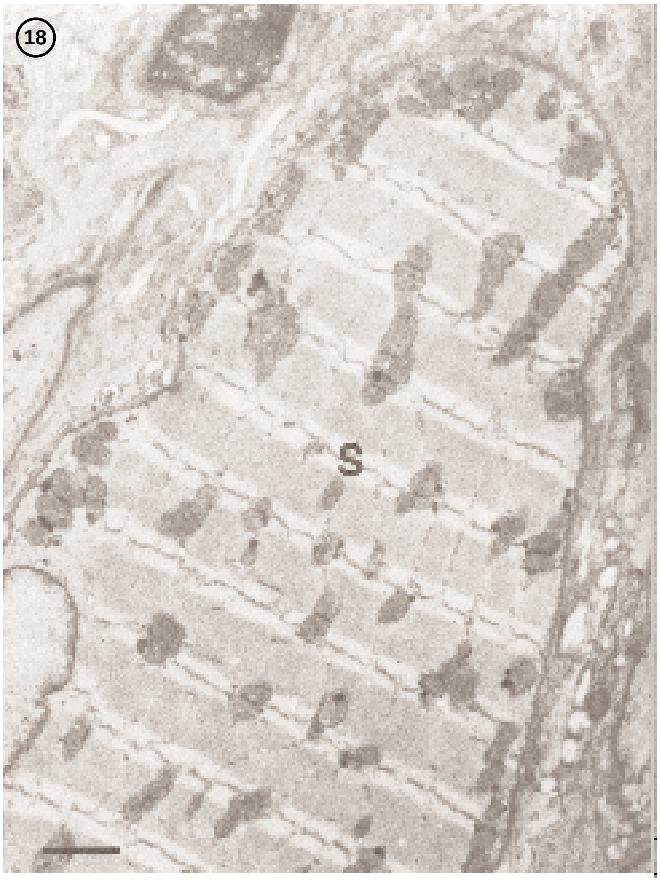

**Figure 17** Electron micrograph of a seminiferous tubule (E) of a 129/SvJ-mouse surrounded by testicular teratoma (T). Residual bodies (arrows) and a thickened basement membrane (Ba) of the seminiferous tubule are seen as a sign of the degeneration caused by tumour compression. The teratoma has grown from the male gonadal ridge of a 12-day-old fetal 129/SvJ-mouse implanted to an adult mouse testis from the same strain for 8 weeks. Bar = 2 μm
**Figure 18** Electron micrograph of a striated muscle cell (S) in testicular teratoma. It has grown from the male gonadal ridge of a 12-day-old fetal 129/SvJ-mouse implanted to an adult mouse testis from the same strain for 11 weeks. Bar = 2 μm

Nine to 12 weeks after implantation the growth of the teratomas had stopped (Figure 16), and they consisted of mature tissue components as shown by electron microscopy (Figures 18–20). In one mouse with experimental testicular teratoma grown for 9 weeks, an extratesticular tumour above the scrotal testicular teratoma was observed. This extratesticular tumour consisted of the same teratomatous tissue as the intratesticular teratoma, and it had a maximum thickness of 8 mm. No distant metastases were observed in the mice with experimental testicular teratomas during 12 weeks after implantation. The characteristics of experimental testicular teratomas cultured for 9–12 weeks have been summarized in Table 2. The frequency of teratomas was 6/20.

### Spontaneous teratomas

One mouse in this material had a unilateral spontaneous testicular teratoma (1/70, 1.5%). It consisted of a large cyst containing serous fluid. In addition, glial cells, respiratory epithelium, striated and smooth muscle and bone marrow surrounded by cartilage and bone could be observed. Very few remnants of seminiferous tubules were observed under the tunica albuginea.

### Immunocytochemistry c-kit, SOX9, GFAP and type IV collagen were localized by immunocytochemistry in three testes with teratomas grown for 4 and 8 weeks. SOX9 was localized in the Sertoli cells of the host testis (Figure 21). In the teratoma it was localized in cartilage cells (only in teratomas cultured for 4 weeks), some epithelial structures and in solitary stromal cells (Figures 22–25). The reaction for c-kit was not observed in the host testis, but only a weak reaction in occasional epithelial structures of teratomas cultured for 8 weeks, and this could not be confirmed by Western blotting. Teratomas were strongly positive for GFAP indicating that glial cells are the predominating cell type of experimental testicular teratomas (Figures 26 and 27), but the host testis was totally negative for GFAP. Type IV collagen demonstrates the histology of a cystic teratoma (Figure 28). Preabsorption of SOX9 antibody with antigen reduced considerably the reaction in positive cells.

### Western blotting

Immunoblotting of GFAP from the testes with teratomas cultured for 7 weeks demonstrated two broad bands with $M_r$ of 30–40 and 90–100 k and immunoblotting for SOX9 demonstrated a band with $M_r$ of 30 k (Figure 29) confirming thus the results of the immunocytochemical reactions.

### DISCUSSION

Experimental testicular teratomas in this study behave invasively inside the embryonic testicular graft by rupturing the embryonic

© Cancer Research Campaign 1999

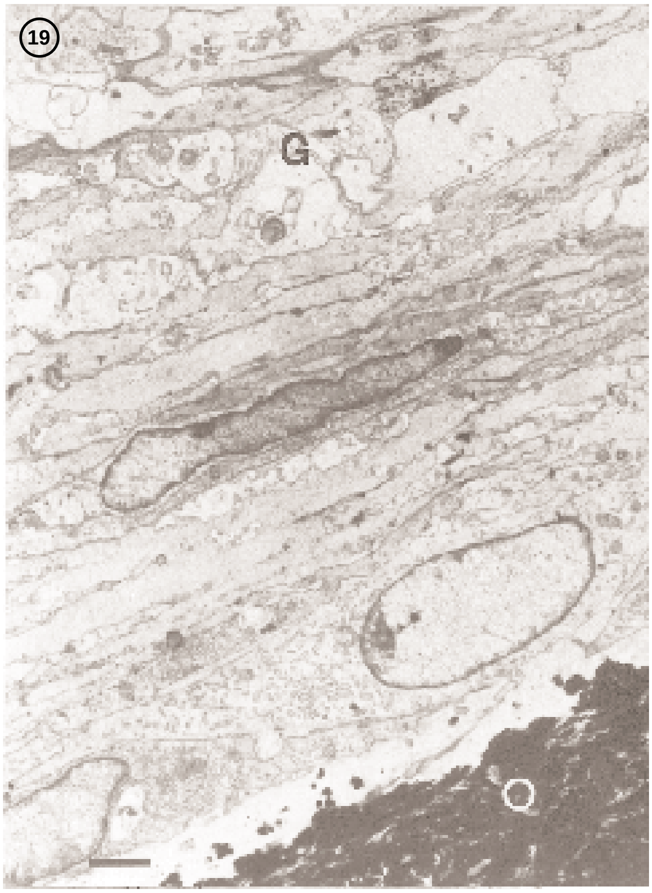

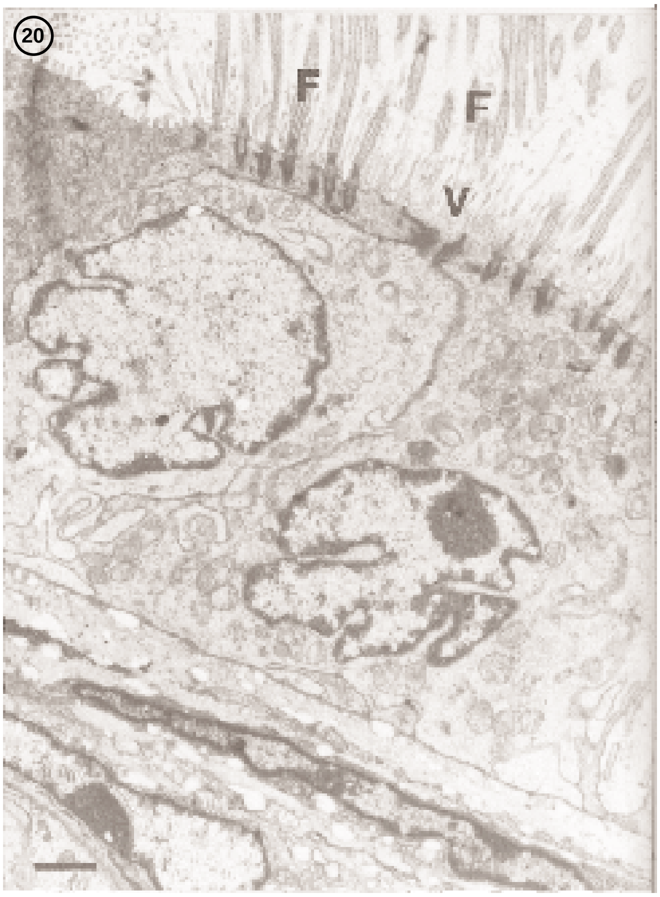

**Figure 19** Electron micrograph of bone (O) and glial cells (G) in testicular teratoma of 129/SvJ-mouse. It has grown from the male gonadal ridge of a 12-day-old fetal 129/SvJ-mouse implanted to an adult mouse testis from the same strain for 11 weeks. Bar = 2 μm
**Figure 20** Electron micrograph of cilia (F) and microvilli (V) in respiratory epithelium of an experimental testicular teratoma of a 129/SvJ-mouse. It has grown from the male gonadal ridge of a 12-day-old fetal 129/SvJ-mouse implanted to an adult mouse testis from the same strain for 8 weeks. Bar = 2 μm

**Table 2** The characteristics of experimental testicular teratomas cultured for 6–12 weeks after implantation of fetal gonadal ridges to adult mouse testes

| Culture time of intratesticular grafts (weeks) | Number of testes with teratomas/ testes per group | Tissue components in teratomas | Size of teratomas |
|---|---|---|---|
| 6–8 | 9/38 | Neural main component with glial cells, glandular structures, striated and smooth muscle, brown adipose tissue, respiratory epithelium, cysts, teratoma with local areas of immature neuroepithelium | Macroscopic enlargement of the scrotum by testicular teratoma, the teratoma has filled the testis except few seminiferous tubules under the tunica albuginea, the mean (s.e.m.[a]) diameter of testes with teratoma in proximal–distal axis: 15 mm (1.1 mm) |
| 9–12 | 6/20 | Neural main component, mature cystic teratoma | Macroscopic enlargement of the scrotum by testicular teratoma, only some remnants of seminiferous tubules of the host under the tunica albuginea, the growth of the teratoma has stopped, the mean (SEM) diameter of testes with teratoma in proximal–distal axis: 11 mm (1.9 mm) |

[a]Standard error of mean.

*© Cancer Research Campaign 1999*

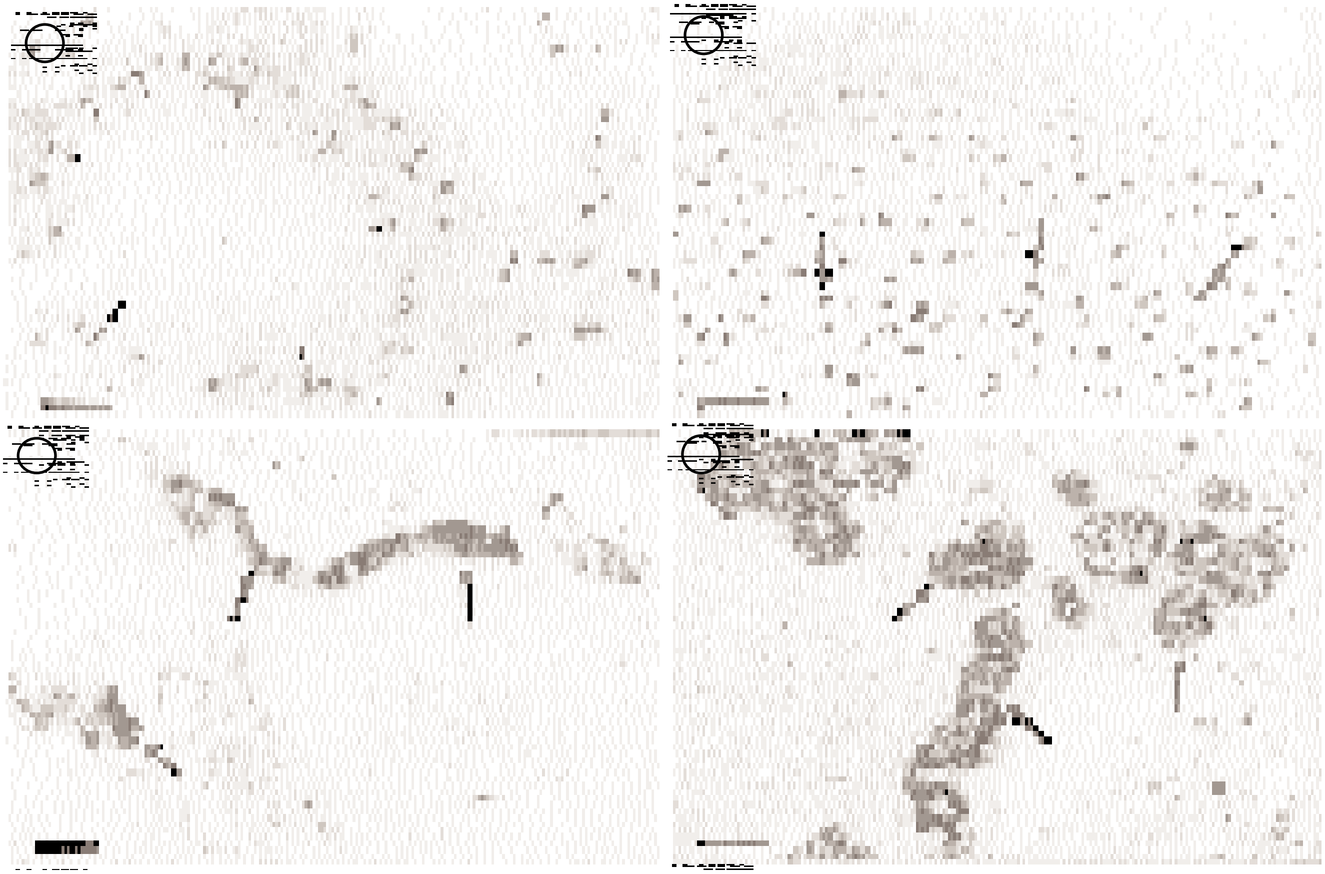

**Figure 21** SOX9 in Sertoli cells of the host testis (arrows) as shown by immunocytochemistry. The teratoma has grown from the male gonadal ridge of a 12-day-old fetal 129/SvJ-mouse implanted to an adult mouse testis from the same strain for 4 weeks. No background staining. Bar = 50 μm
**Figure 22** SOX9 in cartilage (arrows) of teratoma as shown by immunocytochemistry. The teratoma has grown from the male gonadal ridge of a 12-day-old fetal 129/SvJ-mouse implanted to an adult mouse testis from the same strain for 4 weeks. No background staining. Bar = 30 μm
**Figure 23** SOX9 in epithelial structures (arrows) of teratoma as shown by immunocytochemistry. The teratoma has grown from the male gonadal ridge of a 12-day-old fetal 129/SvJ-mouse implanted to an adult mouse testis from the same strain for 8 weeks. No background staining. Bar = 40 μm
**Figure 24** SOX9 in glandular structures (arrows) of an experimental testicular teratoma as shown by immunocytochemistry. The teratoma has grown from the male gonadal ridge of a 12-day-old fetal 129/SvJ-mouse implanted to an adult mouse testis from the same strain for 8 weeks. No background staining. Bar = 30 μm testicular cords and in the host testis by replacing of the host seminiferous tubules by the teratoma, enlarging the testis. However, the tendency of teratoma to spread out from the testis is low during 12 weeks after implantation. One extratesticular tumour with histology of a teratoma was observed in the vicinity of a testicular teratoma, but no distant metastases were observed in this study. However, metastasizing of spontaneous testicular teratomas in the 129/Sv-mice has been described (Stevens, 1973). The extratesticular teratoma of this study may have originated from fetal gonadal ridge cells, which have escaped from the testis during the intratesticular implantation procedure. The low metastasizing capacity and stop of growth 6–8 weeks after implantation of the teratomas in this study might partly be due to gradual apoptotic withdrawal of immature neuroectodermal tissue components as suggested by apoptotic morphology of some of the cells. This suggestion is supported by the relatively small amounts of $^{3}$H-thymidine ($^{3}$HTdR) incorporating cells as observed by in vivo injection of $^{3}$HTdR and autoradiography (unpublished observation). Also most of the spontaneous testicular teratomas of 129/Sv-mice mature completely, but undifferentiated embryonal carcinoma cells have been reported to persist in some animals until adulthood (Stevens, 1958).

Solter et al (1970) have implanted pre-gastrulation mouse embryos of C3H/H-mice to extra-uterine sites, which gave rise to teratocarcinomas, some of which reached an enormous size and killed the host by local expansion, but no metastases were observed in these tumours. Tissue pieces from these tumours and also from spontaneous teratomas with undifferentiated tissue components can be grown in vivo for many years as transplantable tumours (Stevens, 1958, 1970*b*) and in vitro (Finch and Ephrussi, 1967; Martin and Evans, 1974). Controversy still remains about the origins of embryonal carcinoma cells derived from ectopically transplanted embryos (Andrews, 1998), but a variety of data points to similarity of embryonal carcinoma cells to embryonic stem cells of the inner mass and primitive ectoderm in the early mouse embryo (Solter and Damjanov, 1979). In addition, pluripotent human embryonal carcinoma cell lines share many of the typical characteristics identified from human embryonic stem cells in general (Andrews et al, 1996). Indeed, the present model on growth of teratomas from primordial germ cells of grafted fetal gonadal ridges supports the current suggestion that fetal life and infancy play a central role in the genesis of testicular cancer (Stevens, 1967*b*; Van Gurp et al, 1994; Wang and Enders, 1996).

© Cancer Research Campaign 1999

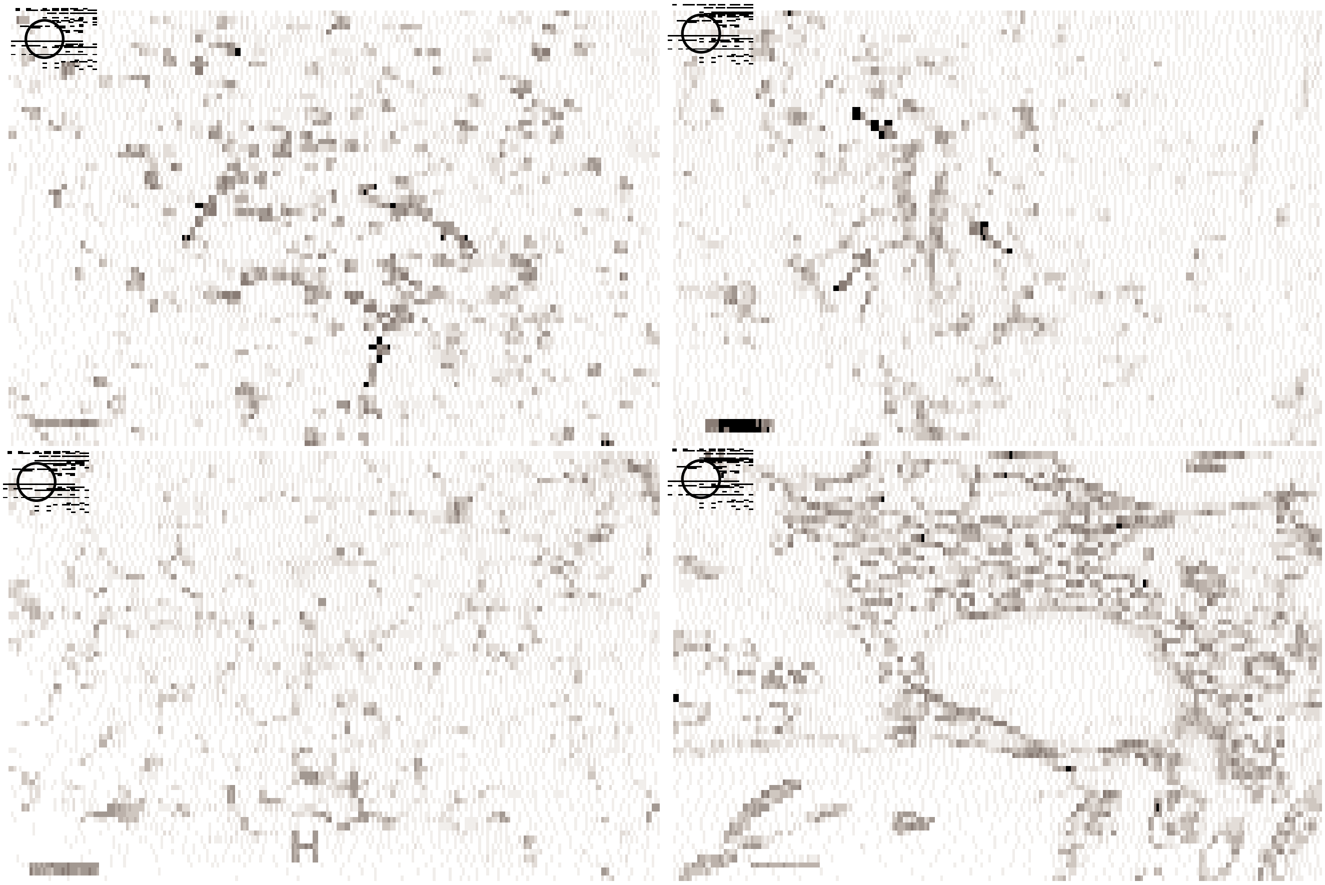

**Figure 25** SOX9 in interstitial cells of a teratoma as shown by immunocytochemistry. The reaction is strictly limited to a part of cells (arrows). The teratoma has grown from the male gonadal ridge of a 12-day-old fetal 129/SvJ-mouse implanted to an adult mouse testis from the same strain for 8 weeks. No background staining. Bar = 30 μm
**Figure 26** GFAP in glial cells (arrows) of a teratoma as shown by immunocytochemistry. The teratoma has grown from the male gonadal ridge of a 12-day-old fetal 129/SvJ-mouse implanted to an adult mouse testis from the same strain for 4 weeks. No background staining. Bar = 20 μm
**Figure 27** GFAP in glial cells of a testicular teratoma as shown by immunocytochemistry, while no reaction is seen in the host testis (H). The teratoma has grown from the male gonadal ridge of a 12-day-old fetal 129/SvJ-mouse implanted to an adult mouse testis from the same strain for 4 weeks. No background staining. Bar = 20 μm
**Figure 28** Type IV collagen in the basement membranes of a teratoma as shown by immunocytochemistry to demonstrate the histology of cystic teratoma. It has grown from the male gonadal ridge of a 12-day-old fetal 129/SvJ-mouse implanted to an adult mouse testis from the same strain for 8 weeks. No background staining. Bar = 50 μm

Both the age of the implanted gonadal ridge and the implantation site are important for induction of teratomas. In earlier studies, germ cells became resistant to the teratocarcinogenic process during the 14th day of gestation (Stevens, 1970*a*, 1973). Our preliminary observations confirm this finding: gonadal ridges from older than 13-day post-coitum (13-dpc) male embryos develop only normal embryonic testis tissue. In the study of Nogushi and Stevens (1982) the decline in the primordial germ cell mitotic activity in older than 13-dpc mouse embryos paralleled the reduced susceptibility to experimentally-induced teratocarcinogenesis. The effect of the implantation site on the induction of teratomas has been studied before (Stevens, 1970*a*), and the scrotal position is highly teratocarcinogenic compared to abdominal sites obviously because of lower temperature in the scrotum (Friedrich et al, 1983). Temperature-dependent expression of susceptibility genes that are yet to be identified might partly explain the connection between temperature and teratocarcinogenesis in 129/Sv-mice (Matin et al, 1998).

In man, non-seminomatous (teratomatous) components of germ cell tumours only develop via a seminomatous stage (Oosterhuis et al, 1989). Instead, rodents develop teratomas directly without an intermediate stage of neoplastic proliferation of primordial germ cells (Walt et al, 1993), and lack carcinoma in situ (CIS) and seminoma. This limits the use of the experimental testicular teratoma in mouse as a tool to understand the genesis of human testicular germ cell tumours. However, this mouse model is useful in the study of experimental damage of the host seminiferous tubules caused by a tumour and in the study of the effects of teratoma on the local immune response of the host (Sundström et al, 1998). The model can also be used to study, how the teratoma reacts to various exogenous stimuli.

In histopathological analysis, the predominance of neural components in experimental testicular teratoma is supported by the presence of many GFAP-positive structures in the teratomas grown for 4 and 8 weeks. Interleukin (IL)-6 and CD1d (lipid antigen presenting molecule) localize in the same neural structures in experimental testicular teratoma as GFAP (Sundström et al, 1998). This suggests a role for glial type cells in immunoregulation of this testicular neoplasm. The immunoregulatory role of human glioma cells is further supported by the observations that

 *© Cancer Research Campaign 1999*

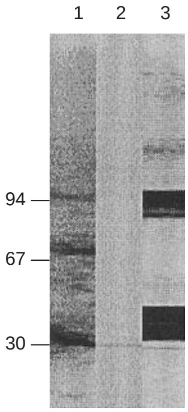

**Figure 29** Immunoblotting of GFAP and SOX9 from the testes with teratomas cultured for 7 weeks. The lanes were as follows: lane 1: standard; lane 2: SOX9, a band with $M_r$ of 30 k; lane 3: GFAP, two broad bands with $M_r$ of 30–40 and 90–100 k these cells express high levels of lymphocyte costimulatory adhesion molecules (especially VCAM-1) in human brain tumours, whereas only very low levels of these molecules were detected in normal brain (Mäenpää et al, 1997) indicating that tumours with glial tissue components have elements to communicate with the host immune system. Interestingly, the human gliomas behave like experimental testicular teratoma: they have local expansive growth and the paucity of distant metastases. The immunologically special characters of these two tumours might play a role in their low metastasizing capacity.

Transcription factor SOX9 localizes only to Sertoli cells as described (Morais da Silva et al, 1996) in the host testis surrounding the graft and in the embryonic testicular graft without teratoma (not presented in this study). In testicular teratomas cultured in vivo for 4 weeks, SOX9 localized to cartilage cells. Localization of SOX9 in cartilage was not observed in teratomas cultured for 8 weeks. It is possible that the chondrocytes in older teratomas matured into hypertrophic cells, because the rapid shutdown of SOX9 expression has been described during this maturation process (Zhao et al, 1997). Indeed, bone and bone marrow, but not cartilage, was seen in teratomas older than 6 weeks. Heterozygous mutations in SOX9 in humans lead to campomelic dysplasia, a severe dwarfism syndrome (Foster et al, 1994), which also poses the important role of this transcription factor in skeleton formation. It has been proposed (Zhao et al, 1997) that SOX9 might also have an important role in neural development, because the expression of this transcription factor has been detected in several neural tissues of the developing embryo, e.g. generation and migration of neural crest cells. In addition to cartilage in teratomas and Sertoli cells in the surrounding host testis, SOX9 localized to several stromal cells of teratomas. We suppose that these SOX9-positive cells in teratomas are of neural origin, because GFAP localized to the same stromal areas as SOX9. It is possible that SOX9 can be used as a marker of the developmental phase of experimental testicular teratoma during the process of differentiation of the immature teratoma into mature tissue components. However, the localization of SOX9 was studied only in teratomas cultured for 4 and 8 weeks, and screening of SOX9 in several time points are needed to confirm this concept.

The localization of c-kit only in occasional glandular structures in teratomas cultured for 8 weeks is in accordance with the previous studies on the localization of c-kit only to CIS-cells and to seminoma, but very seldom to non-seminomas (Rajpert-De Meyts and Skakkebaek, 1994). The CIS-cells and seminoma represent initial stages of germ cell development and the expression of c-kit is obviously lost in non-seminomas, which represent the stages of fetal development.

### CONCLUSIONS

(1) The metastasizing capacity of the experimental testicular teratoma is very low during 12 weeks, but the behaviour of the tumour in the testicular tissue of the graft is invasive. (2) The growth of experimental testicular teratomas cease 6–8 weeks after implantation of the fetal gonadal ridges with the obvious apoptosis of the immature tissue components. (3) The development of testicular teratomas in mouse and human are different, but the present model of experimental testicular teratoma in the mouse is suitable for studying how the teratoma affects the host testis, how the host testis reacts to teratoma and how the teratoma reacts to various exogenous stimuli.

### ACKNOWLEDGEMENTS

The skilful assistance of Mrs Leena Salminen, Mrs Sirpa From, Mr Urpo Reunanen and Dr Jouko Mäki, Ph D, is gratefully acknowledged.

### REFERENCES

Adami HO, Bergström R, Möhner M, Zatonski W, Storm H, Ekbom A, Tretli S, Teppo L, Ziegler H, Rahu M, Gurevicius R and Stengrevics A (1994) Testicular cancer in nine Northern European countries. *Int J Cancer* **59**: 33–38

Andrews PW (1998) Teratocarcinomas and human embryology: pluripotent human EC cell lines. *APMIS* **106**: 158–168

Andrews PW, Casper J, Damjanov I, Duggan-Keen M, Giwercman A, Hata JI, von Keitz A, Looijenga LHJ, Millan LJ, Oosterhuis JW, Pera M, Sawada M, Schmoll HJ, Skakkebaek NE, van Putten W and Stern P (1996) Comparative analysis of cell surface antigens expressed by cell lines derived from human germ cell tumours. *Int J Cancer* **66**: 806–816

Carlsen E, Giwercman A, Keiding N and Skakkebaek NE (1992) Evidence for decreasing quality of semen during past 50 years. *Br Med J* **305**: 609–613

Finch BW and Ephrussi B (1967) Retention of multiple developmental potetialites by cells of a mouse testicular teratocarcinoma during prolonged culture in vitro and their extinction upon hybridization with cells of permanent lines. *Proc Natl Acad Sci USA* **57**: 615–621

Foidart JM, Berman JJ, Paglia L, Rennard S, Abe S Perantoni A and Martin GR (1980) Synthesis of fibronectin, laminin, and several collagens by a liver-derived epithelial line. *Lab Invest* **42**: 525–532

Foster JW, Dominguez-Steglich MA, Guioli s, Kowk G, Weller PA, Stevanovic M, Weissenbach J, Mansour S, Young ID, Goodfellow PN, Brook JD and Schafer AJ (1994) Campomelic dysplasia and autosomal sex reversal caused by mutations in an Sry-related gene. *Nature* **372**: 525–530

Friedrich TD, Regenass U and Stevens LC (1983) Mouse genital ridges in organ culture: the effects of temperature on maturation and experimental induction of teratocarcinogenesis. *Differentiation* **24**: 60–64

Fröjdman K, Pelliniemi LJ and Virtanen I (1998) Differential distribution of type IV collagen chains in the developing rat testis and ovary. *Differentiation* **63**: 125–130

Gilliland FD and Key CR (1995) Male genital cancers. *Cancer* **75**: 295–315

Hsu SM, Raine L and Fanger H (1981) A comparative study of the peroxidase–antiperoxidase method and an avidin-biotin complex method for studying polypeptide hormones with radioimmunoassay antibodies. *Am J Clin Pathol* **75**: 734–738

Karnovsky MJ (1971) Use of ferrocyanide-reduced osmium tetroxide in electron microscopy. *J Cell Biol* **284**: 146

Klepp O, Olsson AM, Henrikson O, Aass N, Dahl O, Stenwig AE, Persson BE, Cavallin-Stahl E, Fossa SD and Wahlqvist L (1990) Prognostic factors in

© Cancer Research Campaign 1999 

clinical stage I non-seminomatous germ cell tumors of the testis: multivariate analysis of a prospective multicenter study. Swedish–Norwegian Testicular Cancer Group. *J Clin Oncol* **8**: 509–518

Martin GR and Evans MJ (1974) The morphology and growth of a pluripotent teratocarcinoma cell line and its derivatives in tissue culture. *Cell* **2**: 163–172

Matin A, Collin GB, Varnum DS and Nadeau JH (1998) Testicular teratocarcinogenesis in mice – a review. *APMIS* **106**: 174–182

McLean IW and Nakane PK (1974) Periodate-lysine-paraphormaldehyde fixative. A new fixative for immunoelectron microscopy. *J Histochem* **22**: 1077–1083

Morais da Silva S, Hacker A, Harley V, Goodfellow P, Swain A and Lovell-Badge R (1996) SOX9 expression during gonadal development implies a conserved role for the gene in testis differentiation in mammals and birds. *Nature Genet* **14**: 62–68

Mostofi FK and Price EB (1973) Tumors of the testis. In *Tumors of the Male Genital System. Atlas of Tumor Pathology,* 2nd series, Fasc. 8, p. 2. Armed Forces Institute of Pathology: Washington DC

Mäenpää A, Kovanen PE, Paetau A, Jääskeläinen J and Timonen T (1997) Lymphocyte adhesion molecule ligands and extracellular matrix proteins in gliomas and normal brain: expression of VCAM-1 in gliomas. *Acta Neuropathol* **94**: 216–225

Noguchi T and Stevens LC (1982) Primordial germ cell proliferation in fetal testis in mouse strains with high and low incidences of congenital testicular teratomas. *J Natl Cancer Inst* **69**: 907–913

Oosterhuis JW, Castedo SMMJ, De Jong B, Cornelisse CJ, Dam A, Sleijfer DT and Schrafford Koops H (1989) Ploidy of primary germ cell tumours of the testis. *Lab Invest* **60**: 14–21

Rajpert-De Meyts E and Skakkebaek NE (1994) Expression of c-kit protein product in carcinoma-in-situ and invasive testicular germ cell tumours. *Int J Androl* **17**: 85–92

Ro JY, Dexeus FH, el-Naggar A and Ayala AG (1991) Testicular germ cell tumors. Clinically relevant pathologic findings. *Pathol Annu* **26**: 59–87

Solter D and Damjanov I (1979) Teratocarcinoma and the expression of oncodevelopmental genes. *Methods Cancer Res* **18**: 277–232

Solter D, Skreb N and Damjanov I (1970) Extra-uterine growth of mouse egg-cylinders results in malignant teratoma. *Nature* **227**: 503–504

Stempak JG and Ward RT (1964) An improved staining method for electron microscopy. *J Cell Biol* **22**: 697–701

Stevens LC (1958) Studies on transplantable testicular teratomas of strain 129 mice. *J Natl Cancer Inst* **20**: 1257–1275

Stevens LC (1964) Experimental production of testicular teratomas in mice. *Proc Natl Acad Sci USA* **52**: 654–661

Stevens LC (1967*a*) The biology of teratomas. In *Advances in Morphogenesis*, Abercrombie M and Brachet J (eds., p. 1–131). Academic Press: New York

Stevens LC (1967*b*) Origin of testicular teratomas from primordial germ cells in mice. *J Natl Cancer Inst* **38**: 549–552

Stevens LC (1970*a*) Experimental production of testicular teratomas in mice of strains 129, A/He, and their F1 hybrids. *J Natl Cancer Inst* **44**: 923–929

Stevens LC (1970*b*) The development of transplantable teratocarcinomas from intratesticular grafts of pre-and postimplantation mouse embryos. *Dev Biol* **21**: 364–382

Stevens LC (1973) A new inbred subline of mice (129/terSv) with a high incidence of spontaneous congenital testicular teratomas. *J Natl Cancer Inst* **50**: 235–239

Stevens LC (1984) Spontaneous and experimentally induced testicular teratomas in mice. *Cell Differ* **15**: 69–74

Stevens LC and Mackensen JA (1961) Genetic and environmental influences on testicular teratocarcinogenesis in mice. *J Natl Cancer Inst* **27**: 443–453

Sundström J, Veräjänkorva E, Salminen E, Pelliniemi LJ and Pöllänen P (1998) Experimental testicular teratoma promotes formation of humoral immune responses in the host testis. *J Reprod Immunol* (in press)

Towbin H, Staehelin T and Gordon J (1979) Electrophoretic transfer of proteins from polyacrylamide gels to nitrocellulose sheets: procedure and some applications. *Proc Natl Acad Sci USA* **76**: 4350–4354

Van Gurp RJLM, Oosterhuis JW, Kalscheuer V, Mariman ECM and Looijenga LHJ (1994) Human testicular germ cell tumours show biallelic expression of the H19 and IGF2 gene. *J Natl Cancer Inst* **86**: 1070–1077

Venable JH and Coggeshall RA (1965) Simplified lead citrate stain for use in electron microscopy. *J Cell Biol* **25**: 407–408

Walt H, Oosterhuis JW and Stevens LC (1993) Experimental testicular germ cell tumorogenesis in mouse strains with and without spontaneous tumours differs from development of germ cell tumours of the adult human testis. *Int J Androl* **16**: 267–271

Wang D and Enders GC (1996) Expression of a specific mouse germ cell nuclear antigen (GCNA1) by early embryonic testicular teratoma cells in 129/Sv-Sl/+ mice. *Cancer Lett* **100**: 31–36

Zhao Q, Eberspaecher H, Lefebve V and De Crombrugghe B (1997) Parallel expression of SOX9 and Col2a1 in cells undergoing chondrogenesis. *Dev Dyn* **209**: 377–386

Zheng T, Holford TR, Ma Z, Ward BA, Flannery J and Boyle P (1996) Continuing increase in incidence of germ-cell testis cancer in young adults: experience from Connecticut, USA, 1935–1992. *Int J Cancer* **65**: 723–729

 *© Cancer Research Campaign 1999*
